# Targeting MICA with therapeutic antibodies for the treatment of cancer

**DOI:** 10.1186/2051-1426-1-S1-P41

**Published:** 2013-11-07

**Authors:** Cécile Bonnafous, Valentine Peri, Sylvia Trichard, Ivan Perrot, Stéphanie Cornen, Ariane Thielens, Violette Breso, Yannis Morel, Benjamin Rossi, Carine Paturel, Laurent Gauthier, Mathieu Bléry

**Affiliations:** 1 Innate Pharma, Marseille, France

## 

MICA and MICB, along with ULPBs, are ligands for the activating receptor NKG2D expressed on NK cells and subsets of T cells in Human. NKG2D ligands are induced by cellular stress and pathogen infections. Their expression is tightly regulated by complex mechanisms both at the mRNA and protein levels. In the case of MICA and MICB, more than 65 and 30 alleles respectively were described with different properties regarding to their cellular location adding to the complexity of this recognition system. Nevertheless, as markers of cellular stress, in particular in tumorigenesis, MICA and the closely related MICB proteins are candidates of choice to be targeted by a cytotoxic therapeutic antibody. We first evaluated MICA/B expression by immunohistochemistry on healthy tissues and tumors to validate these antigens as therapeutic targets. Then, using mouse immunization, we generated a panel of chimeric human IgG1 monoclonal antibodies targeting MICA and MICB. These mAbs have the ability to bind to several structurally different alleles and to cross-react on MIC proteins from cynomolgus macaques. Their capacity to block the MICA/NKG2D interaction was assessed by surface plasmon resonance as well as by using cell-based assays. In vitro efficacy was measured by the capacity to mediate complement-dependent cytotoxicity (CDC) and antibody-dependent cell cytotoxicity (ADCC) towards MICA expressing cells. In vivo efficacy of the anti-MICA mAbs was measured in both a preventive and a curative setting using MICA expressing cell lines. Altogether, we have generated a panel of anti-MICA mAbs with diverse functional properties. Ongoing work aims to choose the best candidate for humanization and further clinical development.

